# Effects and moderators of the Olweus bullying prevention program (OBPP) in Germany

**DOI:** 10.1007/s00787-020-01647-9

**Published:** 2020-09-22

**Authors:** Fanny Carina Ossa, Vanessa Jantzer, Lena Eppelmann, Peter Parzer, Franz Resch, Michael Kaess

**Affiliations:** 1 Department of Child and Adolescents Psychiatry, Centre for Psychosocial Medicine, University Hospital Heidelberg, Blumenstraße 8, 69115 Heidelberg, Germany; 2 Faculty of Behavioral and Cultural Studies, Institute of Psychology, University of Heidelberg, Hauptstraße 47-51, 69117 Heidelberg, Germany; 3 University Hospital of Child and Adolescent Psychiatry and Psychotherapy, University of Bern, Bolligenstrasse 111, Stöckli, 3000 Bern 60, Switzerland

**Keywords:** Bullying, School, Victimization, OBPP, Prevention, Adolescents

## Abstract

Bullying is a common and significant risk factor for mental and physical health problems. The aim of the outlined study was to evaluate the German version of the Olweus Bullying Prevention Program (OBPP) and to investigate potential moderators of its effectiveness. 23 schools started with the implementation and all students were invited to complete the Olweus Bullying Questionnaire annually. For our analyses, the data from grades 5 to 9 were used (t0: *n* = 5759; t1: *n* = 5416; t2: *n* = 4894). 16 out of the 23 schools completed the 18-months implementation period. The effectiveness of the program statistically depended on its complete implementation (*χ*^2^_(2)_ = 7.62, *p* = 0.022). In the group of non-completers, the prevalence of victimization did not change during the observation period of 2 years (*χ*^2^_(2)_ = 4.64, *p* = 0.099). In the group of the completer schools, a significant decrease in bullying between t0 and t1 was found for victims (t0: 9.14%; t1: 6.87%; OR = 0.74; 95% CI 0.62–0.88; *p* = 0.001) and perpetrators (t0: 6.16%; t1: 4.42%; OR = 0.70; 95% CI 0.55–0.89; *p* = 0.004). After 24 months (t2), this decrease could be retained (victims: t2: 6.83%; OR = 0.73; 95%CI = 0.61–0.88; *p* = 0.001; perpetrators: t2: 4.63%; OR = 0.72; 95% CI 0.57–0.92; *p* = 0.009). Furthermore, we found the following moderators of program effectiveness in the completer schools: (1) gender (with a stronger decrease among victimized girls; *p* = 0.004) and (2) school grade (with a stronger decrease of victimization among grades 5–7; *p* = 0.028). The German version of the OBPP significantly reduced the bullying prevalence in the completer schools. Effective prevention needs time and resources: fulfilling the 18-months implementation period was the basis for positive results.

**Electronic supplementary material:**

The online version of this article (10.1007/s00787-020-01647-9) contains supplementary material, which is available to authorized users.

## Introduction

Bullying is defined as negative actions, which occur repeatedly and over a long period of time. It is discerned from peer-conflicts by an imbalance of power. Consequently, the person who is being bullied has difficulties to defend him-/herself and the perpetrator is superior [1]. The large-scale study “Health Behavior in School-Aged Children” (HBSC) with a sample of over 200,000 adolescents from 40 European countries estimates the worldwide prevalence of bullying victimization with 12.6%, ranging across countries from 4.8% to 45.2% [2]. In most countries, the rates of victimization decreased with age. The data on the association of victimization and gender are inconsistent so far. However, boys were more likely to be perpetrators than girls. The prevalence for being a perpetrator is estimated at 10.7% [2]. In Germany, the recent studies reported prevalence rates of victimization between 10 and 16% [3, 4].

Bullying increases the risk for a wide and diverse range of health and psychosocial problems with long-term effects even in later adulthood [5, 6]. Furthermore, our group recently reported significantly higher healthcare costs among victims of repetitive bullying compared to children without any bullying experience [7]. Despite its serious negative outcomes, bullying is still the most pervasive form of aggression at school [8]. Although it appears to be part of everyday school life, it is often disregarded. Even though many teachers recognize bullying in their schools, they often feel helpless or not responsible. Some schools have established prevention strategies, but most of them have failed to document and evaluate results. Considering the fact that school is mandatory in many parts of the world and students consequently have to spend a lot of time at school, schools must ensure that every student is safe at school and does not develop consequential impairments [1, 6]. Therefore, effective and long-term bullying prevention is urgently needed [9].

In a meta-analytic review, Ttofi and Farrington [10] investigated the effectiveness of 44 anti-bullying programs at schools. Being bullied decreased by 17–20% on average, being a perpetrator by 20–23%. More intensive and long-lasting programs with a whole-school policy were more effective and “programs inspired by the work of Dan Olweus worked best”. In a more recent meta-analysis [11], the Olweus Bullying Prevention Program (OBPP) still worked best in reducing perpetrators and was among the best methods in reducing victimization. Professor Dan Olweus developed the OBPP, an evidence-based prevention program which had its beginnings in Scandinavia in 1983 [12]. Two large Norwegian evaluation studies of the OBPP showed a relative decrease in bullying of 24–43% on the side of the victims and a relative decrease of 21–52% for perpetrators in grade 5–7. A 5-year follow-up study in Oslo revealed that this decrease could be retained for both victims and perpetrators [12]. Positive results have also been reported for students in grades 8–10, but less consistently and sometimes weaker [12]. The OBPP spread out during the recent years and also had a large implementation period in the United States. A current study evaluated the program in 210 schools in Pennsylvania using an age cohort design [13]. Almost all grades showed significant reductions over 2 years with higher decreases for the lower grades (relative reduction: victims: 8.2–19.2%; perpetrators: 29.7–34.6%). Limber et al. [13] additionally found a program by sex interaction for students in grades 3–5. For boys they found a greater reduction for being victimized than for girls. For being a perpetrator, no gender effects were found. Other studies reported equal changes in bullying among boys and girls [12]. A first attempt to implement the OBPP in Germany dates back to 1994 [14]. The study was inspired by the book “Bullying at school, what we know and what we can do” [15]. The research team implemented their version in 37 German schools and evaluated it with *N* = 11,052 students. The results showed a reduction in direct victimization in grades 3, 5, 6 and 7, but not for indirect victimization. Perpetrator rates decreased in grades 4, 5 and 7. Grades 11 and 12 showed an increase in bullying. According to Olweus and Limber [16] the program “deviated considerably […] from the OBPP in terms of program components and model of implementation”. Therefore, our study can be regarded to be the first to implement the OBPP in Germany.

The aim of the current study was to evaluate the implementation of the German version of the OBPP in a sample of German secondary schools. We hypothesized that the number of bullying victims and perpetrators will be reduced when schools successfully implement the OBPP. In addition, potential moderator effects of gender, class level and school-type were tested.

## Methods

### Study population, procedures and design

The German OBPP project was funded by the Baden-Wuerttemberg Foundation (Baden-Wuerttemberg Stiftung) as part of their program “Youths Mental Health”; thus, participation in the OBPP was free of charge for all schools. The study was conducted at the Department of Child and Adolescent Psychiatry, University of Heidelberg and performed in compliance with the Helsinki Declaration. It was approved by the ethics committee of the Medical Faculty of the University of Heidelberg (S-341/2014) and the respective school authorities. In addition, the study was registered at a WHO trial registry (Deutsches Register Klinischer Studien; DRKS00008202).

A prospective quasi-experimental design with an annual student survey (baseline, 12 months follow-up, 24 months follow-up) was used, and the OBPP was implemented after baseline in each participating school. All students, teachers and caregivers were informed about the OBPP implementation as well as the evaluation study by leaflet. Regarding the survey, students gave informed consent and caregivers were informed about the study and had the opportunity to object to the participation of their child (opt-out). The survey was conducted annually just before summer vacation. The data were collected from students in class-sized groups during a 45 min online survey.

The recruiting process started in 2014. Only regular secondary schools (i.e., no schools for students with special needs; no part time or evening schools) with at least 100 students, starting from grade five, were eligible. The intended design was a randomized control trial (RCT) and 30 schools from our regional catchment area were randomly selected and invited to participate. Although the schools were intensively contacted via both mail and personal phone call, all of these schools declined. Subsequently, the catchment area was expanded stepwise. It took schools between several weeks and months to settle their decisions, and we still received very little consent to participate, thwarting the project timeline and funding resources. Given that we were forced to work with a lower number of schools than originally expected, and that schools were even less willing to participate in the context of a potential allocation to a control group, we finally moved from the RCT design to a quasi-experimental evaluation study.

Initially, 413 secondary schools in the state of Baden-Wuerttemberg were informed about the program via e-mail, mail and phone calls, and invited to participate in the OBPP and its evaluation. 13 schools accepted our offer and started with the program in 2015 (first cohort). In a second step, we further expanded the catchment area and invited another 1102 schools (794 new schools and 308 schools who did not respond during the first cohort). 10 schools accepted our invitation and started the program in 2016 (second cohort). Supplement figure A1 provides detailed information about participants in each group following the CONSORT 2010 Flow Diagram [17]. Overall, 195 (16.1%) out of the 1210 invited schools signaled interest and made closer contact via telephone, e-mail or in person. 23 schools finally started the program, resulting in a recruitment rate of 1.9% (see supplement figure A1). These schools were defined as the intention to treat (ITT) schools in our study, out of which 16 finally finished the implementation period (completer schools). In the participating schools (*n* = 23), a total number of *n* = 6485 students (grades 5–9) were invited to complete the survey. The final number of students who consented to participate in the OBPP evaluation during baseline assessment was *n* = 5759 (88.8% response rate). We divided grades 5, 6 and 7 (age 10–13 years) and grades 8–9 (age 14–16 years) into two different grade groups.

### The German Olweus bullying prevention program

Prior to the start of the study, all OBPP materials were translated into German in close collaboration with Olweus International Bergen and a translation company. The original teacher handbook was translated from Norwegian [18], the parent’s booklet was translated from English [19] and the Olweus film was dubbed from Norwegian into German. Our study was the first implementation and evaluation of the OBPP in the German school system with the original materials; nonetheless, some minor changes and adaptions to the original were necessary and can be found in the supplementary material (supplement table A1).

For every school, a keyperson, called Olweus-Coach, was selected. This person received a 7 day training carried out by Olweus International Bergen (3 blocks: (1) day1–3: before implementation started, at the beginning of the school year, (2) day 4/5: 5 months after the program had started, (3) day 6/7: at the end of the school year), as well as regular supervision during the implementation phase. The Olweus-Coach in turn was responsible for the implementation of the program components at school, for example regular study- and supervision groups for all staff members, regular class meetings and cooperation with parents. For further details of the program, see Olweus and Limber [12] and supplementary table A2. Participation in three student surveys, as well as active work with the program and the Olweus-Coach over a period of at least 18 months were defined as minimum criterion for the program’s complete implementation. Active work included small groups for the teacher staff at least every 6 weeks, discussions and regular contact with the Olweus-Coach, restructuring of the supervision system, regular classroom lessons at least in grades five through eight and further program components (see Supplement Table A2). Regular telephone calls (every 3 months), between the research team and the Olweus-Coaches made sure that the core components of the program were running according to the Olweus manual. In addition, there was an annual call with the headmaster of each school.

### Measures

The Olweus Bullying Questionnaire Revised (OBQ-R) [20] is a 57-item questionnaire that anonymously assesses students’ self-reports of bullying others and being bullied at school or via electronic means (cyberbullying), their own behavior when they witness bullying, their attitudes towards bullying and their perceptions of how their teachers counteract bullying [21, 22]. The two global items “How often have you been bullied/have you bullied others at school during the past 3 months?” were used to define victims and perpetrators with the common cut-off of “at least 2 or 3 times a month”.

### Statistical analyses

The effect of the intervention on the rate of bullying was estimated with multilevel mixed-effect logistic regressions with being bullied (yes/no) as dependent variable and time of assessment (t0, t1 and t2) as fixed factor. To take into account that students within one class are more similar then students between classes and students within one school are more similar than students between schools we added random intercepts for each school and for each combination of class and assessment time nested within schools. The short-term effect (1 year) of the program is estimated by the odds ratio between the probability of being bullied at t0 and t1 and the long-term effect (2 years) by the odds ratio between t0 and t2. We also tested the possible moderator variables program completion, gender, grade-level, school-type and cohort. For each of these moderator variables the variable itself and the interaction of the variable with assessment time were added to the regression. A significant interaction would mean that the program effect is influenced by the levels of the moderator variable. Of special interest is cohort as moderator. A significant interaction between assessment time and cohort would indicate a confounding between program effect and general trends in time, limiting the interpretation of the results. To estimate the effect of the program on the number of bullying perpetrators, the same set of models were estimated with being a bullying perpetrator (yes/no) as dependent variable. We assumed an independent variance–covariance structure for all mixed models; the hypotheses were tested with Wald-tests of linear contrasts using a significance level of α = 0.05. For all calculations, the statistic program Stata 16 was used [23].

## Results

Out of the 23 participating schools (intention-to-treat), 16 schools successfully implemented the OBPP (completer), while seven schools canceled implementation of the program within the 18-months implementation period (non-completer). Five of these seven non-completers took part in the annual student survey at all three time points and two schools completed the survey at two time points only. Table 1 presents the sample description for completer and non-completer schools as well as the overall sample.

**Table 1:** Sample description

	Total	Completer	Non-completer
Number of schools	23	16	7^b^
*N*			
t0	5759	4753	1006
t1	5416	4466	950
t2	4894	4305	589
Gender (%)			
Girls	47.79	48.77	42.55
Boys	52.21	51.23	57.45
Grade-level (%)			
5–7	56.76	57.05	55.21
8–9	43.24	42.95	44.79
School-type (%)^a^			
A	26.78	31.82	0
B	73.22	68.18	100
Participation (%)	88.11	90.20	78.45

^a^*Gymnasium* is called A-level school; *Haupt-* and *Realschule* were summarized as B-level schools

^b^Two of the seven non-completer schools did not participate in t2

### Intent-to-treat analysis and effects in the non-completer schools

At baseline, 9.21% reported being a victim of bullying, with a range of 2.0–17.46% among the 23 schools. An intent-to-treat analysis (including both completers and non-completers) showed a significant reduction between t0 and t1 (t1: 7.13%; relative reduction 22.56%; OR = 0.80; 95% CI 0.65–0.98; *p* = 0.033) but no reduction between t0 and t2 (t2: 7.55%; relative reduction 17.99%; OR = 0.99; 95% CI 0.79–1.24; *p* = 0.948). 6.71% reported being a perpetrator, with a range of 0.0–16.39% across schools. The number of perpetrators was reduced between t0 and t1 (t1: 4.99%), which comes up to a relative reduction of 25.62% (OR = 0.71; 95% CI 0.56–0.92; *p* = 0.010). Again, this effect was not stable at t2 (t2: 5.06%; relative reduction: 24.58%; OR = 0.79; 95% CI 0.59–1.04; *p* = 0.093).

**Fig. 1:**
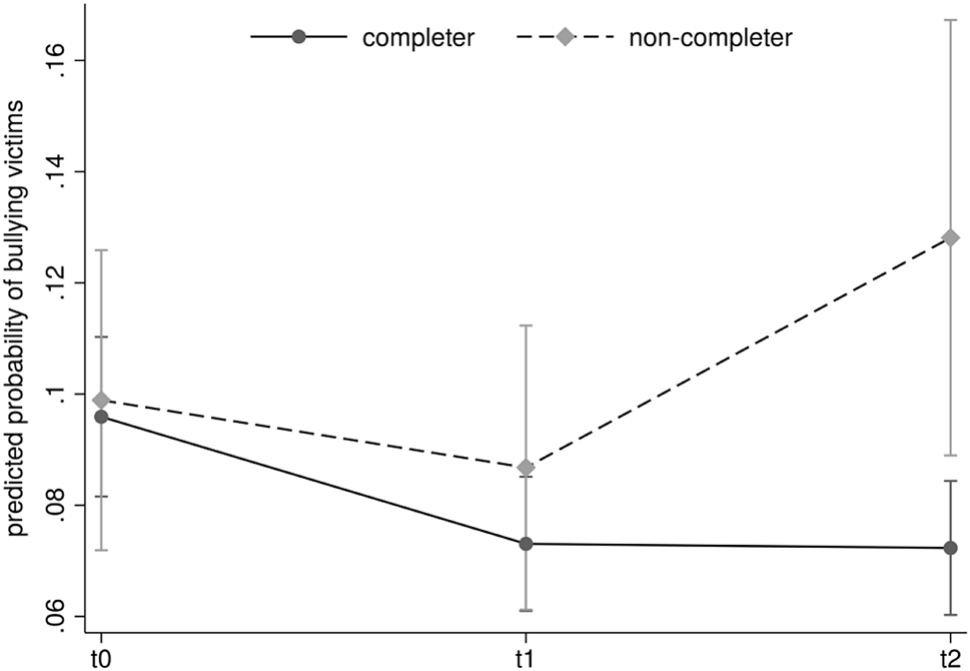
Predicted probability of victimization (%) at baseline (t0), 12-months follow-up (t1) and 24-months follow-up (t2), separated for the completer and non-completer schools

A significant interaction between program participation and assessment time was found in the regression of bullying victimization (*χ*^2^_(2)_ = 7.62, *p* = 0.022), indicating that the trajectories of the victimization rates differed between completer and non-completer schools. While the completer schools showed a significant reduction in victimization over time (*χ*^2^_(2)_ = 15.17, *p* < 0.001), the non-completers showed no change in the rate of victimization during the observation period (*χ*^2^_(2)_ = 4.64, *p* = 0.099). Figure 1 shows the predicted probability of victimization for all time points, separately for completer and non-completer schools. For perpetrators, no interaction between program participation and assessment time was found (*χ*^2^_(2)_ = 0.35, *p* = 0.840).

To make meaningful conclusions about the effect of the OBPP, its complete implementation is the basic requirement. Therefore, the following analyses refer to the 16 completer schools only.

### Effects in the completer schools

At baseline, 9.14% reported being a victim of bullying. After 1 year, the bullying rate dropped to 6.87% at t1 (OR = 0.74; 95% CI 0.62–0.88; *p* = 0.001). This corresponds to a relative reduction of 24.87% in being bullied. Between t0 and t2, the relative reduction remained 25.26% (rate at t2: 6.83%; OR = 0.73; 95% CI 0.61–0.88; *p* = 0.001), indicating stable program effects. These odds ratios reflect a small effect size in epidemiological studies [24].

The number of perpetrators could be reduced from 6.16% at t0 to 4.42% at t1, which corresponds to a relative reduction of 28.25% (OR = 0.70; 95% CI 0.55–0.89; *p* = 0.004).The reduction was stable at t2 (rate at t2: 4.63%; relative reduction: 24.86%; OR = 0.72; 95% CI 0.57–9.2; *p* = 0.009). These results reflect small effect sizes [24].

### Moderators of the program effect

#### Gender

At t0, no differences for the prevalence of victimized girls (9.75%) and boys (8.54%; *z* = − 1.76; *p* = 0.078) were found. Including gender as a moderator of the program effect resulted in a significant interaction between gender and assessment time for victims (*χ*^2^_(2)_ = 10.85; *p* = 0.004). Girls showed a significant decrease between t0 and t1 (OR = 0.66; 95% CI 0.52–0.84; *p* = 0.001), as well as between t0 and t2 (OR = 0.55; 95% CI 0.42–0.71; *p* < 0.001). This indicates that being a victim of bullying for girls reduced by half after 2 years of intervention, which is interpreted as a medium effect size in epidemiological studies [24]. Boys showed no change over the course of the program (t0 vs. t1: OR = 0.83; 95% CI 0.65–1.05; *p* = 0.125; t0 vs. t2: OR = 0.93; 95% CI 0.74–1.18, *p* = 0.570).

**Fig. 2:**
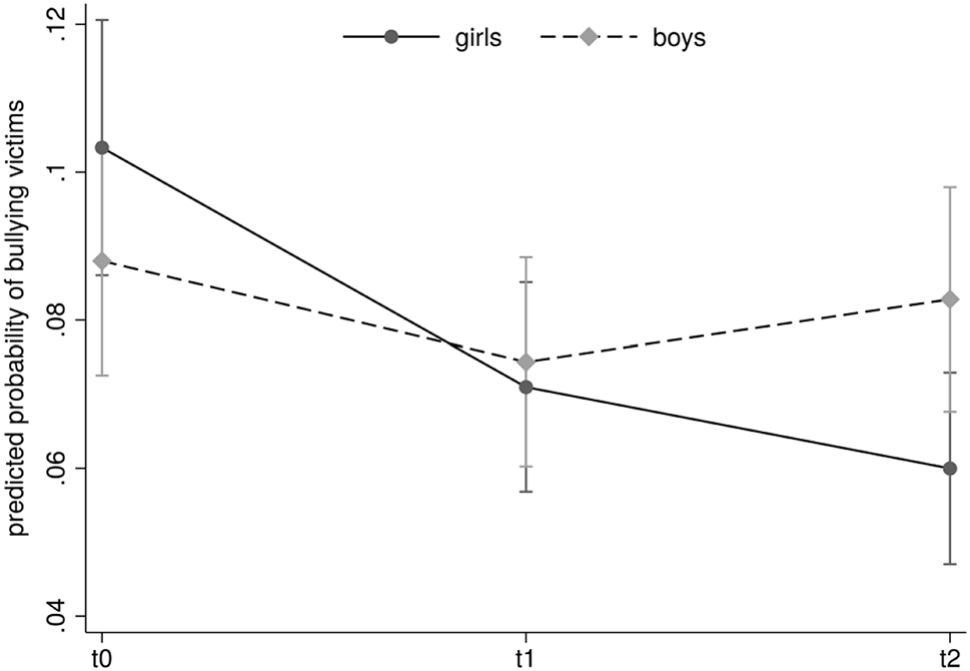
Predicted probability of victimization (%) at baseline (t0), 12-months follow-up (t1) and 24-months follow-up (t2), separated by gender for the completer schools

At t0, boys were significantly more likely to be perpetrators than girls (boys: 8.61%; girls: 3.70%; OR = 2.47; 95% CI 1.90–3.22; *p* < 0.001). As opposed to victims, there was no interaction between gender and assessment time for perpetrators (*χ*^2^_(2)_ = 0.68; *p* = 0.713), indicating that the program did not affect male and female perpetrators differently. Table 2 presents detailed data for bullying and gender. Figure 2 shows the predicted probability of being a victim at all measuring points separated for boys and girls.

**Table 2:** Prevalence (%) and relative change (%) in being bullied and in bullying others over time for the completer schools

	Victims					Perpetrators				
	t0	t1	t2	t0–t1	t0–t2	t0	t1	t2	t0–t1	t0–t2
Gender										
Boys	8.54	7.11	7.95	16.76	6.91	8.61	6.14	6.65	28.62**	22.67*
Girls	9.75	6.61	5.61	32.13**	42.44***	3.70	2.60	2.41	29.64	34.90*
Grade-level										
5–7	10.00	7.79	6.43	22.05*	35.74***	5.29	3.91	3.53	26.14	33.41*
8–9	8.07	5.62	7.40	30.41**	8.36	7.23	5.10	6.15	29.42*	14.97
School-type										
A	7.51	6.08	7.04	19.01	6.30	5.25	3.79	5.46	27.84	-4.00
B	9.92	7.26	6.74	26.87**	32.02***	6.60	4.73	4.27	28.29*	35.25**
Total	9.14	6.87	6.83	24.87**	25.26**	6.16	4.42	4.63	28.25**	24.86**

^*^p < .05; **p < .01; ***p < .001

#### Grade-level

At t0, an inverse relationship between the prevalence of being bullied and grade was found, with a victimization rate of 10.00% at grades 5–7 and 8.07% at grades 8–9 (OR = 0.78; 95% CI 0.61–0.99; *p* = 0.044). The data showed a significant interaction between grade group and assessment time in predicting victimization (*χ*^2^_(2)_ = 7.12; *p* = 0.028). Grades 5–7 showed a significant decrease between t0 and t1 (OR = 0.77; 95% CI 0.61–0.97; *p* = 0.025) and between t0 and t2 (OR = 0.62; 95% CI 0.49–0.79; *p* < 0.001), which is a medium effect size in epidemiological studies [24]. Grades 8–9 showed a significant decrease between t0 and t1 (OR = 0.68; 95% CI 0.51–0.92; *p* = 0.009; medium effect size) but there was no change between t0 and t2 anymore (OR = 0.91; 95% CI 0.69–1.20; *p* = 0.507).

At t0, the overall rate of perpetrators differed between the grade groups, with higher rates in grades 8–9 (grades 5–7: 5.29%; grades 8–9: 7.23%; OR = 1.41; 95% CI 1.03–1.92; *p* < 0.032). For being a perpetrator, no interaction of assessment time with grade group could be shown (*χ*^2^_(2)_ = 1.25; *p* = 0.535); see Table 2 for further details.

#### School-type

At t0, no differences for the prevalence of victimization in A-level (7.51%) and B-level schools (9.92%) were found (*z* = 1.60; *p* = 0.109). There was no interaction between school-type and assessment time in predicting victimization (*χ*^2^_(2)_ = 3.13; *p* = 0.209).

At t0, the prevalence for being a perpetrator did not differ between A-level (5.25%) and B-level (6.60%) schools (*z* = 0.62; *p* = 0.538). Also, the interaction between school-type and assessment time was not significant in predicting perpetration (*χ*^2^_(2)_ = 4.88; *p* = 0.087). Again, see Table 2 for further details.

#### Cohort

There was no interaction between cohort and assessment time for victims (*χ*^2^_(2)_ = 5.61; *p* = 0.061) and for perpetrators (*χ*^2^_(2)_ = 3.69; *p* = 0.158), indicating that the program did not affect the two cohorts differently.

## Discussion

In the light of the adverse long-term effects bullying has on victims and perpetrators and the increasing need for successful prevention, the purpose of this study was to evaluate the effects of the German OBPP. The results provide support for its effectiveness among participating schools. Within the completer sample, there were clear reductions in the two key dimensions, being victimized and being a perpetrator. The reductions were visible after 1 year in both groups and were even maintained after 2 years. In contrast, non-completer schools had no reduction of the bullying rates during the observation period. In addition, there was no cohort-effect on bullying reduction, which indicates that the results were not driven by general time trends. This supports the assumption that the bullying reduction of the completer schools is likely facilitated by program implementation and that prevention “programs need to be intensive and long lasting to have an impact”, as Ttofi [10] has already concluded.

The bullying reduction among victims and perpetrators after 1 year was 24.87–28.25%, considered as small effect size in epidemiological studies [24]. The reduction is generally consistent with previous research evaluating the OBPP in the US or Norway [10, 13, 16]. In their current meta-analysis, Gaffney, Farrington and Ttofi [11] estimated an approximate reduction of 15–16% for bullying victimization and 19–20% for bullying perpetration over 100 evaluations. By comparison, the reported reduction by implementing the German OBPP seems to be above average. A positive effect could be retained for victims and perpetrators after 2 years, with a relative reduction of bullying of 25%. This is in line with Gaffney, Farrington and Ttofi [11], who claimed the OBPP to be the most effective intervention program in reducing school-bullying perpetration. Gaffney, Farrington and Ttofi [11] also identified global differences in the effectiveness of anti-bullying programs. Five German evaluation studies met the pre-determined inclusion criteria, as described in the meta-analysis [11]: studies must “(1) describe an evaluation of a school-based anti-bullying program that was implemented with school-age participants; (2) utilize an operational definition of school bullying that coincides with common definitions; (3) measure school-bullying perpetration and/or victimization using quantitative measures; and (4) use an experimental or quasi-experimental design […]”. These five evaluations revealed a significant reduction of victimization (Odds Ratio 1.18), but not of perpetration. Again, the German OBPP appears promising.

Our study revealed two moderator effects which shall be briefly discussed here. First of all, the program effect in Germany was obviously stronger for girls (42.44% reduction, medium effect size), than for boys which might suggest that the program is more effective in reducing female victimization. However, it is also well possible that girls may tend to answer more socially desirable in respective outcome questionnaires. This was rather surprising since no previous studies working with the OBPP reported an interaction by gender in this direction. Therefore, it would be questionable to explain this effect only by program content or requirements that may favor girls. So far, authors reported that some components of anti-bullying programs worked better for girls (e.g., monitoring school break times), others for boys (e.g., clear rules or disciplinary strategies; [25]). It is possible that our participating schools put special emphasis on components such as improved break supervision and thus reductions in bullying rates were higher for girls. Smith et al. [25] concluded that the success of targeted prevention and intervention factors may differ between girls and boys and may therefore not be universal. To explain gender differences in further detail, the different types of bullying (verbal, social, physical and cyber) should be investigated separately because some involve boys more frequently than girls [26]. The program might also evoke stronger compassion for victimized girls than for boys because of biased gender role stereotypes that promote toughness as an especially masculine trait [27]. Kochenfelder-Ladd and Skinner [28] reported that seeking social support reduced the risk for peer victimization for girls, whereas seeking social support was associated with low peer acceptance for victimized boys. Boys earn respect of their peers by handling peer conflicts themselves. Detecting boys in need for support becomes thus even harder for teachers and parents, while for these boys appealing for help would certainly undermine their peer status even further. Scheithauer et al. [26] reported the important role of gender composition within class: bullying occurred more frequently in classes with mainly boys. In follow-up studies, this class composition factor should be considered as control factor. Considering the behavior of boys in more detail would help to identify aspects that could reduce male victimization. In addition, teacher ratings would be helpful to exclude a social desirability effect.

Secondly, the victimization rate could be reduced between t0 and t1 in both grade-levels; however, after 2 years, the positive effects could only be retained for grades 5–7 but not for grades 8–9. Thus, we revealed an overall successful pattern for grades 5–7, with a medium effect size [24], but a different pattern for grades 8–9: a strong reduction in being a victim as well as a perpetrator between t0 and t1 (around 30%, medium effect size), but no significant effect after 2 years anymore. This finding was unexpected and warrants further investigation. It is also not in line with the previous results from Norway and the US, where program effects at higher grade levels have been somewhat weaker and have taken longer time to obtain [13, 16]. An analysis from Olweus and Kallestad [29] about the effective implementation of the classroom aspects showed that more of the low-grade teachers had used at least one of the effective classroom measures as compared to high-grade teachers. Limber et al. [13] mentioned that school structures change in the higher grades (e.g., less time with classroom teacher), which could make it more difficult to address bullying. In our study, schools started approximately 3 months before t1 with classroom components in most grades. It is possible that schools had a good start in all grade levels, explaining the immediate effect, but could not maintain the regularity or intensity in the higher grades during the second year of program implementation. A detailed dosage–response analysis could help to explain this grade effect in the future. Another aspect is the decrease of bullying with increasing age, as we found at the baseline. This finding confirms previous research [2, 26] and emphasizes the fact that there was a higher need for the program in the lower grades, what has certainly influenced program activity in turn.

Despite the significant reduction of bullying in the completer schools, the study demonstrates a potential lack of feasibility of the OBPP in the current German school system. As illustrated by our difficulties in recruitment, German schools have been unlikely to participate in the OBPP even under relatively optimal conditions (i.e., no program costs or close support and supervision from the research team). In the future—outside of research studies like ours—schools would have to pay for the materials and Coach-Workshops, which may even increase barriers for the successful implementation of the program. Thus, the overall public health value of the OBPP for Germany may be questionable. In this study, we provided transparent reporting of our difficulties in recruiting schools including its implications for further dissemination. Four studies conducted in Germany during the last decade were included in a recent meta-analysis. However, none of these provided detailed reporting of recruitment rates [11, 30]. The main international studies were conducted under different conditions (i.e., nationwide campaigns with mandatory participation), not allowing for a direct comparison [1, 31]. Only one German study from 1994, inspired by OBPP, reported a recruiting rate of 4.45%, which is likewise low [14]. The sample sizes of the four German studies (published post 2009), included in the recent meta-analysis [30], varied between *n* = 119 and *n* = 422. Therefore, we consider the large sample size (of *N* = 5759) and our transparency in the reporting of the recruitment process as two major strengths, when compared with the previous studies. Our findings of low feasibility may point to the need of new programs that integrate effective whole-school approaches but require less time and costs to be implemented. However, prevention definitely needs time and resources to be effective [10] and it is probably unrealistic to expect that bullying prevention can achieve large effect sizes for very low costs and efforts. Therefore, and particularly since bullying is associated with significant costs to society [7], an increase of resources for prevention efforts within the school system might also be a potential solution to the problem.

Consequently, our conclusion is: It is necessary to increase the feasibility of the OBPP or any other antibullying program that may include similar whole-school approaches probably alongside with political actions that may also involve the provision of respective resources. If schools work with the OBPP over a period of 18 months, they may experience considerable benefits as indicated by reduced bullying rates.

### Limitations

For interpreting our results, it needs to be considered that these are not the results from a RCT. Although an RCT was originally planned, the study design was changed due to the low commitment of schools to participate in the study in general, and in a control condition in particular (see Figure A1 of the supplementary material). It is not new that the realization of a RCT design in complex organizations like schools is problematic [13]. However, the intent-to-treat analyses made it possible to compare completer and non-completer schools and therefore control for some influencing and irrelevant factors like initial motivation to engage in bullying prevention as well as general time trends. However, RCTs still remain the gold standard, and are mainly lacking in bullying intervention and prevention research. Another limitation may be related to both the representativeness of the schools as well as the overall practicability of the OBPP in Germany. The required resource-neutral implementation of the OBPP resulted in the massive recruitment difficulties as described above. Most decisions at German schools are democratically based. In order to participate in a whole-school program, the majority of teachers have to vote for it in a teachers´ conference. Although 16.12% of all invited schools signaled serious interest and need for bullying prevention, only 1.90% finally decided to partake. The low recruitment rate shows that the majority of the schools were deterred by a time-consuming program like the OBPP. Hence, the study demonstrates a potential lack of feasibility of the OBPP in the current German school system. The final sample of our study likely is a group of highly motivated schools and hence may not be entirely representative for the German school system, which is also reflected in the fact that the reported baseline rates of victimization (9.14%) were below the national average of 10–16% [3, 4]. This sample bias may thus reduce the generalizability of the study. However, given that we were forced to broaden our catchment area substantially due to low consent rates among the originally invited schools, the recruitment strategy was in many cases limited to sending personal letters via mail or e-mail to all schools. Improved personalized and proactive recruitment strategies might have been successful in improving the participation rate; however, these are costly and time consuming and might even attenuate ecological validity of the study. Political campaigning to raise general awareness of the problem might further guide these efforts and ease initial contact to schools. In addition, one third of the schools dropped out during the first year. Different from the completer schools, the non-completer sample consisted of B-level schools only which potentially led to further selection bias (overrepresentation of A-level schools among completers). Although bias could not be controlled for within our study design, this bias may be partly alleviated by our finding that school types did not differ in their bullying reduction rates within the completer group. Main reasons given for quitting the program were too little time or motivation, high turnover of staff, transfer of leadership or different priorities and school profiles. Challenges in disseminating the OBPP are not new and have already been discussed by Olweus and Limber [16]. This raises the question for possible program adaptations, making it easier for the schools and staff to begin with and maintain the program. Possible adaptations could be a lower frequency in regular teacher meetings through online alternatives like chats and e-learning; focusing on grades five through eight, where the highest bullying rates occur and where teachers have greater flexibility. At higher grades fewer class meetings or special theme-days would be conceivable. A wider variety of pre-structured materials for the use in class meetings might further aid in limiting preparation time for teachers. Further studies into the efficacy of components could help design and optimize a program that better values existing resources. Structural changes within the school system and more flexibility in program implementation could offer chances for the OBPP in the future. Without appropriate political actions, it is likely that only few schools will be willing to go the extra mile to generate substantial change in the future. School authorities should therefore prioritize bullying prevention since obligatory nation-wide campaigns have proven successful, as described earlier. Greene [8] pleads for framing bullying in a human rights approach. He argues that a positive climate change can emerge if state-wide or national bullying laws are invoked as part of a broader human rights perspective. This would lead to a conceptualization of bullying as a form of abuse, where the absence of a clear bullying policy can be seen as a failure to render assistance.

### Further research

Bullying reduction can be achieved by the German OBPP in case of a successful implementation of the program over a period of 18 months. However, the extremely low participation rate outlined above as well as the high dropout rate during implementation (both at the school level), imply that some necessary adaptations might be needed to make the program more feasible for schools. Moderator analyses showed that adaptations may also be needed to make the bullying prevention more effective for boys and more stable for grades eight through nine. In their meta-analysis, Gaffney, Farrington and Ttofi [11] reported that an Italian online prevention program (NoTrap!) worked best at reducing victimization. Including an online forum, short video clips and more materials for class meetings might be promising complements to the traditional work of the OBPP.

## Electronic supplementary material

Below is the link to the electronic supplementary material.

Supplementary file1 (DOCX 418 kb)
