# Supplementary material for: Effects and moderators of the Olweus bullying prevention program (OBPP) in Germany
Source: Eur Child Adolesc Psychiatry. 2020 Sep 22;30(11):1745–54. doi: 10.1007/s00787-020-01647-9 (PMC8558185; doi:10.1007/s00787-020-01647-9)
Supplement: Supplementary file 1 — Supplementary file1 (DOCX 418 kb) [file 787_2020_1647_MOESM1_ESM.docx]

**Effects and moderators of the Olweus Bullying Prevention Program (OBPP) in Germany**

**Journal: [European Child & Adolescent Psychiatry](https://www.springer.com/journal/787)**

Fanny Carina Ossa^12^, Vanessa Jantzer^1^, Lena Eppelmann^1^, Peter Parzer^1^, Franz Resch^1^ & Michael Kaess^13*^

^1^Department of Child and Adolescents Psychiatry,

Centre for Psychosocial Medicine,

University Hospital Heidelberg,

Blumenstraße 8, 69115, Heidelberg, Germany

²Faculty of Behavioral and Cultural Studies,

Institute of Psychology,

University of Heidelberg,

Hauptstraße 47-51, 69117, Heidelberg, Germany

^3^University Hospital of Child & Adolescent Psychiatry and Psychotherapy

University of Bern,

Bolligenstrasse 111, Stöckli, 3000 Bern 60, Switzerland

*Corresponding author:

Prof. Dr. med. Michael Kaess

Fon: +41 31 932 85 52

[michael.kaess@upd.ch](mailto:michael.kaess@upd.ch)

**Supplement tables and figures**

**Supplement figure A1:** Flow diagram of recruitment and selection of participating schools.

| **Supplement table A1** |  |  |
| --- | --- | --- |
| Cultural adaptation of implementation process (18 months) | | |
|  | Original OBPP (Olweus, 2015b; Olweus, 1994; Olweus & Limber, 2010; Olweus & Limber, 2009) | Pilot Project Germany |
| Study- and supervision groups (all staff) | Norway: every other week for all staff, 90 minutes  US: every month, 45 minutes | Approximately once a month for 90 minutes; at least 75% of the staff |
| Class meetings | Once a week in every class | As much as possible (most schools could offer regular class meetings for grade 5-8 only); some schools provided class meetings just once a month |
| Kick-off event for all students | Obligatory | Optional |
| Training of Olweus-Coaches | Norway: 11-12 whole days divided into three appointments over a period of 18-24 months  US: 5 whole days divided into two appointments; phone calls with a mentor once a month over a period of 18-24 months | 3 days of workshop in month 1 (at the beginning of the school year); 1 half day of video-supervision in month 3; 2 days of workshop in month 6; 2 days of workshop in month 10; 1 half day of video-supervision in month 15; every third month telephone calls with the research team |

| **Supplement** **table A2** | |
| --- | --- |
| Components of the OBPP |  |
| School-Level | Establish a Prevention Committee |
|  | Conduct Committee and staff training |
|  | Administer the OBQ schoolwide |
|  | Hold staff discussion group meetings |
|  | Introduce the school rules against bullying |
|  | Review and refine the school´s supervisory system |
|  | Hold a school kick-off event to launch the program |
|  | Involve parents |
| Classroom-Level | Post and enforce schoolwide rules against bullying |
|  | Hold regular class meetings |
|  | Hold meetings with students´ parents |
| Individual-Level | Supervise students´ activities |
|  | Ensure that all staff intervene on the spot when bullying occurs |
|  | Conduct serious talks with students involved in bullying |
|  | Conduct serious talks with parents of involved students |
|  | Develop individual intervention plans for involved students |
| Adaptation from Olweus & Limber, 2009 | |
